# Cytotoxic Activity of *Origanum Vulgare* L. on Hepatocellular Carcinoma cell Line HepG2 and Evaluation of its Biological Activity

**DOI:** 10.3390/molecules22091435

**Published:** 2017-08-30

**Authors:** Hazem S. Elshafie, Maria F. Armentano, Monica Carmosino, Sabino A. Bufo, Vincenzo De Feo, Ippolito Camele

**Affiliations:** 1 School of Agricultural, Forestry, Food and Environmental Sciences, University of Basilicata, Potenza 85100, Italy; hazem.elshafie@unibas.it; 2 Department of Sciences, University of Basilicata, Potenza 85100, Italy; mariafrancesca.armentano@unibas.it (M.F.A.), monica.carmosino@unibas.it (M.C.), sabino.bufo@unibas.it (S.A.B.); 3 Department of Pharmacy, University of Salerno, Salerno 84084, Italy; defeo@unisa.it

**Keywords:** anticancer therapeutic agents, GC-MS analysis, liver tumor cell line, renal cell line, antibacterial activity, phytotoxic activity

## Abstract

The potential of plant essential oils (EOs) in anticancer treatment has recently received many research efforts to overcome the development of multidrug resistance and their negative side effects. The aims of the current research are to study (i) the cytotoxic effect of the crude EO extracted from *Origanum vulgare* subsp *hirtum* and its main constituents (carvacrol, thymol, citral and limonene) on hepatocarcinoma HepG2 and healthy human renal cells HEK293; (ii) the antibacterial and phytotoxic activities of the above EO and its main constituents. Results showed that cell viability percentage of treated HepG2 by EO and its main constituents was significantly decreased when compared to untreated cells. The calculated inhibition concentration (IC_50_) values for HepG2 were lower than healthy renal cells, indicating the sort of selectivity of the studied substances. Citral is not potentially recommended as an anticancer therapeutic agent, since there are no significant differences between IC_50_ values against both tested cell lines. Results showed also that oregano EO and its main constituents have a significant antibacterial activity and a moderate phytotoxic effect. The current research verified that oregano EO and its main constituents could be potentially utilized as anticancer therapeutic agents.

## 1. Introduction

There is no doubt that the development of multidrug resistance in cancer therapy, their serious side effects, as well as their high costs have stimulated new recent cancer therapy approaches to find out new, more efficacious and less toxic treatments.

Essential oils (EOs) are one of the most important natural products derived from plants, due to their various biological properties and their medicinal and nutritional uses [1]. Many plant EOs have been used as medicine for centuries and have demonstrated several health benefits, including effects on infectious, chronic, and acute diseases [2]. Numerous EOs have shown antimicrobial activity against several plant and human pathogens [3,4].

Moreover, some EOs have acquired a great potential interest, being able to successfully treat different types of cancer cells. EOs from *Melissa officinalis* L., *Melaleuca alternifolia* (Maiden & Betche) Cheel, *Salvia officinalis* L., *Thymus vulgaris* L., and *Origanum vulgare* L. are examined for their cytotoxic and antiproliferative actions in tumor cell lines to evaluate their efficiency as possible alternatives for cancer treatments [5,6].

The genus *Origanum* (family *Lamiaceae*) includes 39 species widely distributed in the Mediterranean region [7,8]. *O. vulgare* is a perennial herbaceous plant, known commonly as “oregano”, with four subspecies in Italian flora.

Numerous studies have focused recently on the active secondary metabolites of this plant [9]. Oregano EO displayed antimicrobial and antiviral activities [10,11,12,13]. Mancini et al. [14] studied the chemical composition of oregano EO from different regions in Italy and reported the presence of several constituents with promising antifungal activity against *Monilinia laxa* (Aderh. & Ruhland) Honey, *M. fructigena* Honey, and *M. fructicola* (G.Winter) Honey. Moreover, oregano EO and its components significantly inhibited some phytopathogenic fungi such as *Botrytis cinerea* Pers., *Penicillium italicum* Wehmer, *P. expansum* Link, *Phytophthora citrophthora* (R.E. Sm. & E.H. Sm.) Leonian and *Rhizopus stolonifer* (Ehrenb.: Fr.) Vuill. [15] and *Aspergillus niger* Tiegh., *A. flavus* Link, *A. ochraceus* K. Wilh., *Fusarium oxysporum* W.C. Snyder & H.N. Hansen, *F. solani* var. *coeruleum* (Mart.) Sacc., *Penicillium* sp., *Pseudomonas aeruginosa* J.Schrt. ATCC 2730, *Staphylococus aureus* Rosenbach ATCC 6538, *Clavibacter michiganensis* S., *P. infestans* Mont., *Sclerotinia sclerotiorum* Lib., and *Xanthomonas vesicatoria* Doidge [16].

Several studies stated that *O. vulgare* ssp. *hirtum* EO is mainly composed of phenols, *p*-cymene, and γ-terpinene [17]. Mockute et al. [18] mentioned that linalyl acetate, *β*-caryophyllene and sabinene are the principal constituents of *O. vulgare* EO, whereas, D’Antuono et al. [19] and Perez et al. [20] reported that linalool is the main volatile component of oregano EO. Recently, the chemical composition of the EO extracted from *O. vulgare* ssp. was reported by Mancini et al. [14] and the results explicated that the phenolic compounds thymol and carvacrol were the major constituents of oregano EO, followed by linalool and *trans*-caryophyllene.

El Babili et al. [21] reported the cytotoxicity of ethyl acetate and ethanol extracts from leaves of *O. compactum* against human breast cancer cells (MCF7). On the other hand, Bakkali et al. [22] and Mezzoug et al. [23] reported that carvacrol, the major component of oregano EO, showed antimutagenic activity, which seems to be mainly linked to the induction of mitochondrial dysfunction.

The main objectives of this study are to assess (i) the cytotoxic effects of oregano EO and its main constituents against hepatocarcinoma cell line HepG2. Healthy human renal cells HEK293 were used as a control to test the putative selectivity of EO and chosen compounds; (ii) the antibacterial activity of the above EO and its main constituents against *Bacillus megaterium* de Bary ITM100 gram positive (G+ve), *B. megaterium* Planch. (Act.) (G+ve) and *Escherichia coli* Migula gram negative (G−ve); iii) the phytotoxic effect of the studied EO against two common sensitive plant species (*Raphanus sativus* L. cv. “Saxa” and *Lepidium sativum* L.) and two weed species (*Sinapis arvensis* L. and *Phalaris canariensis* L.).

## 2. Results

### 2.1. Chemical Composition of Oregano EO

Hydrodistillation of the aerial parts of *O. vulgare* gave a yellow-reddish oil in 1.0% yield. Table 1 reports the composition of the *O. vulgare* EO; compounds are listed according to their elution order on an HP-5MS column.

In all, 71 compounds were identified, accounting for 91.4% of the total oil. Phenolic fraction (77.2%) dominates in the oil, with the complex carvacrol/thymol accounting for 74.8%. Monoterpenes represent 5.9% of the oil, being monoterpene hydrocarbons 1.7%, with limonene (1.3%) as the main representative of this class. Oxygenated monoterpenes constitute 4.2% of the oil, and (E)-citral is the main component (2.5%). Thirty-seven sesquiterpenes were identified in the EO, representing 7.3% of the total oil. Among sesquiterpenes hydrocarbons (5.8%), germacrene A (1.1%), and *δ*-cadinene (1.0%) predominates, whereas *α*-cadinol (0.4%) and caryophyllene oxide (0.3%) are the main constituents of oxygenated monoterpenes (1.5%).

### 2.2. Cytotoxic Effect of Crude Oregano EO on HepG2 and HEK293 Cells

#### 2.2.1. MTT Assay

**Figure 1:**
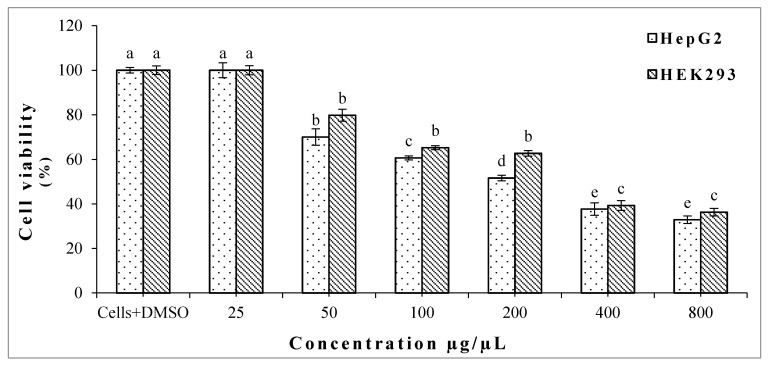
Viability percentage of HepG2 and HEK293 after treatment of crude oregano Essential Oils (EO). Bars with different letters for each cell line indicate mean values significantly different at *p* < 0.05 according to Tukey (B) test. Data are expressed as mean of three replicates ± SD.

In this study, the putative cytotoxic effect of oregano EO against two human cell lines was investigated using a MTT viability assay. As shown in Figure 1, the crude EO extract has significantly reduced the cell viability of the hepatocarcinoma cell line (HepG2) in dose dependent mode (studied range 25–800 µg/µL), as compared to the vehicle control. Interestingly, results of the MTT assay showed that oregano EO has lower cytotoxic activity against non-tumor cell line HEK293 (Figure 1), especially in the range between 100 to 800 µg/µL, as confirmed by the IC_50_ values (236 µg/µL for HepG2 cells, and 310 µg/µL for HEK293 cells). These preliminary results drew our attention to screen the potential cytotoxicity of the main constituents of oregano EO against the same tested cell lines to determine the most effective substance.

#### 2.2.2. Observation of Morphological Changes

**Figure 2:**
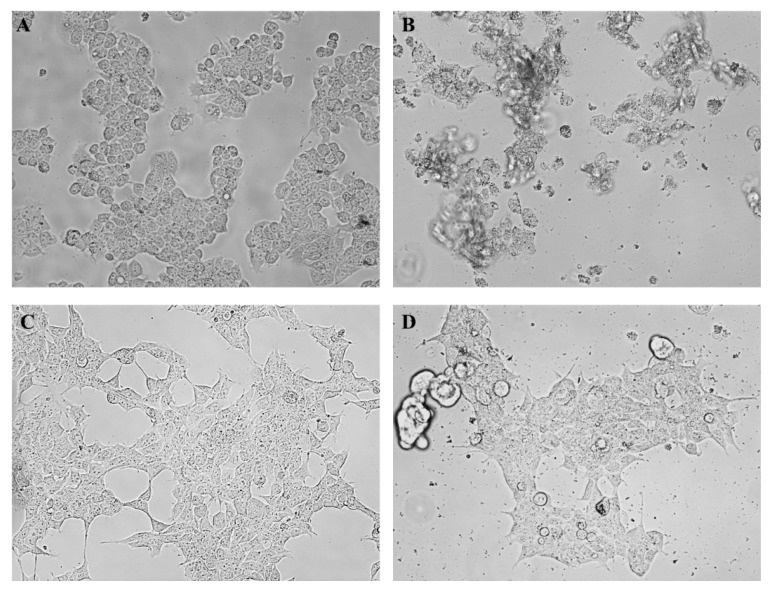
Effect of crude oregano EO on both HepG2 and HEK293 cell morphology. The photographs were taken at a magnification × 40. Images are representative of three independent experiments. Where: (**A**) HepG2 (control); (**B**) HepG2 cells treated with oregano EO (236 µg/µL); (**C**) HEK293 (control) and (**D**) HEK293 cells treated with oregano EO (236 µg/µL).

The effect of oregano EO treatment (24 h) on HepG2 and HEK293 cell morphology were evaluated using inverted phase contrast microscopy. Both types of cells were treated using the IC_50_ value calculated in the case of hepatocarcinoma cells (236 µg/µL). Control cells (treated only with dimethyl sulfoxide (DMSO)) show the normal morphology (Figure 2A,C), whereas, HepG2 cells showed remarkable morphological changes, such as detaching in the degradation phase (Figure 2B). HEK293 cells were less affected and demonstrated lower morphological changes (Figure 2D).

### 2.3. Cytotoxic Effect of Single Substances on HepG2 Cells

#### MTT Assay

**Figure 3:**
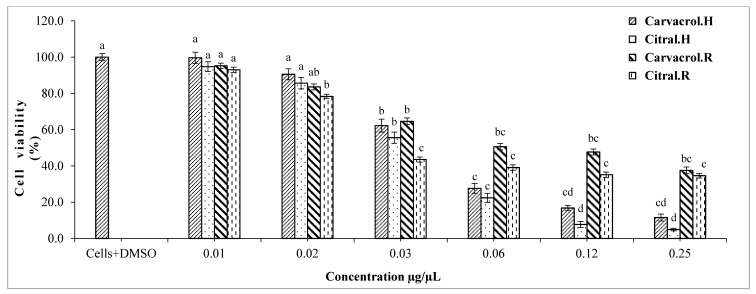
Viability percentage of HepG2 and HEK293 cells after treatment with different concentrations of citral and carvacrol. Bars with different letters for each single substance indicate mean values significantly different at *p* < 0.05 according to Tukey (B) test. Data are expressed as mean of three replicates ± SD. Where: carvacrol.H: liver cells treated with carvacrol; citral.H: liver cells treated with citral; carvacrol.R: renal cells treated with carvacrol; citral.R: renal cells treated with citral.

**Figure 4:**
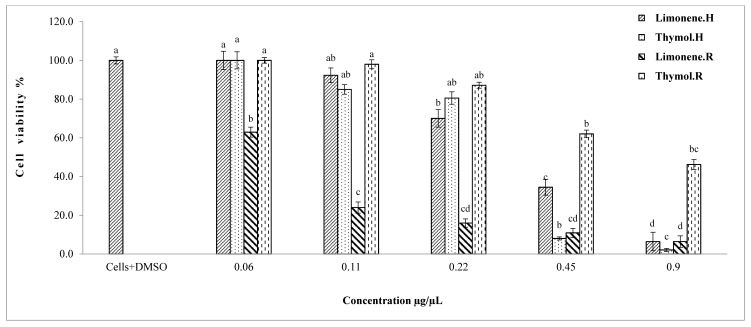
Viability percentage of HepG2 and HEK293 cells after treatment of thymol and limonene. Bars with different letters for each single substance indicate mean values significantly different at *p* < 0.05 according to Tukey (B) test. Data are expressed as mean of three replicates ± SD. Where: carvacrol.H: liver cells treated with carvacrol; citral.H: liver cells treated with citral; carvacrol.R: renal cells treated with carvacrol; citral.R: renal cells treated with citral.

Results of the cytotoxicity test of the main single constituents explicated that carvacrol and citral showed the highest significant reduction of cell viability of hepatocarcinoma cells in the range from 0.01 to 0.25 µg/µL, respectively (Figure 3). In particular, IC_50_ values were 48 and 35 mg/L, respectively, for carvacrol and citral (Table 2). Thymol and limonene showed a viability reduction of HepG2 cells in the range from 0.06 to 0.90 µg/µL (Figure 4), and their IC_50_ values were 289 and 294 mg/L, respectively (Table 2).

### 2.4. Cytotoxic Effect of Single Constituents on HEK293 Cells

#### MTT Assay

The IC_50_ values of renal cells that had been treated with carvacrol and citral were 90 and 32 mg/L, respectively, (Table 3) as compared to 48 and 35 mg/L, respectively, in the case of hepatocarcinoma cells (Table 2). Moreover, IC_50_ values were evaluated for thymol and limonene as 940 and 120 mg/L, respectively, in the case of renal cells (Table 3), as compared to 289 and 294 mg/L, respectively, in the case of hepatocarcinoma cells (Table 2). Generally, the renal cell viability was estimated by 35% and 38% in the case of treatment with citral and carvacrol at 0.25 µg/µL, respectively (Figure 3). Whereas, the hepatocarcinoma cell viability showed lower values estimated by 5% and 12% in the case of the same treatment (Figure 3). In addition, 46% of the renal cells were viable when treated with thymol at 0.90 µg/µL (Figure 4), as compared to hepatocarcinoma cells where only 2% were viable when treated with the same dose (Figure 4). In the case of limonene, there are no significant differences between the cell viability of renal cells and hepatocarcinoma cells, where the highest tested dose of limonene (0.90 µg/µL) explicated about 6% viability in the case of both studied cell lines.

### 2.5. Observation of Morphological Changes 

**Figure 5:**
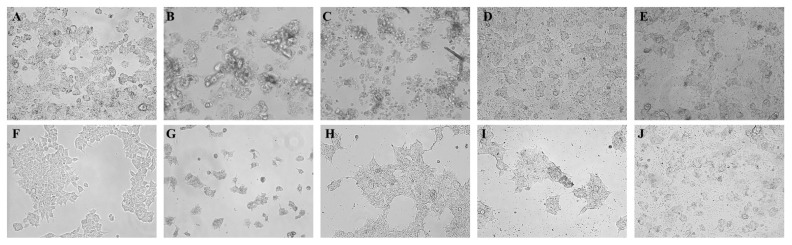
Effect of single pure substances on HepG2 and HEK293 cells morphology. The photographs were taken at a magnification ×40. Images are representative of three independent experiments. Where **A** and **F**: HepG2 and HEK293 (control); and, **B**, **C**, **D** and **E** are HepG2 cells treated with limonene 294 mg/L, thymol 289 mg/L, carvacrol 48 mg/L, citral 35 mg/L, respectively. Whereas **G**, **H**, **I** and **J** HEK293 cells treated with the same previous doses (limonene 294 mg/L, thymol 289 mg/L, carvacrol 48 mg/L, citral 35 mg/L, respectively).

The effect of single purified substances, extracted from oregano EO were evaluated on both HepG2 and HEK293 cell morphology by using the calculated IC_50_ values previously reported for HepG2 as following: limonene 294 mg/L, thymol 289 mg/L, carvacrol 48 mg/L, and citral 35 mg/L. The morphological changes were observed using phase-contrast microscopy. The control HepG2 and HEK293 cells (treated only with DMSO) showed the normal morphology (Figure 5A,F).

Results demonstrated that about 50% of hepatocarcinoma cells treated with limonene 294 mg/L became shrunk and degraded (Figure 5B), whereas, about 70–80% of renal cells were degraded by using the same dose (Figure 5G). In the case of thymol treatment at 289 mg/L, about 50% of the hepatocarcinoma cells were degraded (Figure 5C), while there was no alteration of cell morphology or any indication for toxicity against renal cells using the same dose of thymol (Figure 5H). On the other hand, the treatment of carvacrol at 48 mg/L caused the degradation of about 50% of hepatocarcinoma cells (Figure 5E), as compared to the healthy renal cells, which explicated high viability estimated by 70% (Figure 5I). Finally, the treatment of citral at 35 mg/L showed the degradation of both HepG2 and HEK293 cells by 50% without significant differences between them (Figure 5E,J).

### 2.6. In Vitro Antibacterial Assay

The obtained results showed that the studied EO and its main single substances were able to inhibit significantly the growth of tested G+ve bacterial strains more than G−ve ones in a dose dependent manner (Table 4). In particular, the growth of both tested *B. megaterium* strains were significantly reduced by the tested EO and all single substances. In addition, citral showed the highest bactericidal effect against all tested bacteria especially at 50% concentration. However, the EO and all tested single substances exhibited low activity against the growth of *E. coli*.

### 2.7. Phytotoxic Activity Assay

The studied crud oregano EO was evaluated for its phytotoxic activity on the germination and radicle elongation of two common sensitive species (*R. sativus* and *L. sativum*) and two weed species (*S. arvensis* and *P. canariensis*) (Table 5).

Generally, oregano EO showed a moderate phytotoxic effect against all tested plant species. In particular, the studied EO affected the germination and radical elongation in a different way. At doses of 0.625 and 0.125 μg/mL, oregano EO significantly inhibited the seed germination of *R. sativus* (Table 5). At 0.250 and 0.125 μg/mL, the studied EO significantly inhibited the seed germination of *L. sativum.* The germination index in the case of *P. canariensi* was significantly reduced at 0.062 μg/mL of oregano EO.

## 3. Discussion

The composition of EO agrees partially with our previous studies, in which the EO of the same species presented the same chemical pattern [14] except for the presence of limonene and E-citral. The EO belongs to the carvacrol/thymol chemotype. In fact, *Origanum* taxa can be divided in three chemotypes: (i) linalool, terpinen-4-ol, and sabinene hydrate group; (ii) carvacrol and/or thymol group); and (iii) sesquiterpene group [24]. Other authors proposed the existence of four chemotypes (thymol, carvacrol, thymol/carvacrol and carvacrol/thymol) of *O. vulgare* grown in Calabria (Southern Italy) [17]. The EO and its main components (carvacrol, thymol, limonene and citral) have been evaluated for their possible antiproliferative activity.

The results demonstrated the selective action of carvacrol and thymol against tumor cells rather than healthy renal cells.

The traditional chemotherapy methods for treating the cancer cells rely mostly on the characteristics of drugs to reduce the ability of cancer cells to continuously grow, multiply, and finally to induce the cell damage and death. For these reasons, some interesting natural alternatives have acquired a great potential interest, such as EOs, which are able to successfully treat different types of cancer cells.

Generally, the possible mechanisms of the neoplastic transformation of normal healthy cells could be explained by remaining the signaling of proliferation, inactivating the growth suppressors, and finally by facing the cell death [25]. The potential efficacy of tested oregano EO is thought to depend on the specific toxicity of their single main active constituents and/or by their synergic effect [26,27,28], Grbović et al. [28] concluded that oregano is a significant source of biologically active substances that have cytotoxic activity against colon (HCT-116) and breast cancer (MDA-MB-231). In addition, Sivropoulou et al. [29] reported that oregano EO exhibited high cytotoxicity against four permanent animal cell lines including two lines derived from human cancers. In particular, oregano EO caused complete cell death in all cell lines tested with dilutions of up to 1/10,000 and its ID_50_ on Vero cells was 1/36,000 [29].

The possible expected mechanism of the cytotoxicity effect for either oregano EO or its main single constituents, especially citral, carvacrol, and thymol, could be due to the induction of the activity of Glutathione S-Transferase (GST) in various tissues. GST is thought to play an important role in detoxifying chemical carcinogens [26]. Several studies have also examined the microscopic morphological changes for a variety of cells treated with different substances by using inverted a phase contrast microscope [30,31,32] to understand the possible mechanism of the cytotoxicity effect. The obtained results of the microscopic morphological observations from this study underlined that the possible mechanism of the cytotoxicity of oregano EO and its main single constituents could be due to the induction of cell death by apoptosis and/or necrosis, which leads to increasing the cell permeability and hence losing several cytoplasmic organelles. Furthermore, the lipophilic nature and low molecular weights of the single constituents of studied oregano EO may ascribe their ability to immigrate easily through the cell membrane and alter its phospholipid layers which leads to increasing the fluidity of the cell membrane and finally destroying the cytoplasmic content [25,33].

On the other hand, the IC_50_ values of both citral and limonene against renal health cells were estimated by 32 and 120 mg/L, as compared to 35 and 294 mg/L in the case of hepatocarcinoma cells. These results determine that these two single substances are not highly recommended as potential anticancer therapeutic agents. These observations agree with De Martino et al. [34,35], who concluded that citral is considered as an apoptotic inductor both in leukocytes of healthy subjects and chronic myeloid leukemia patients [36]. In addition, citral and some of its related compounds showed significant in vitro toxicity against pancreatic cell tumor lines (MIA PaCa2 cells) and human B-lymphoma as reported by Di Mola et al. [37].

The antibacterial activity can be explained by the chemical structure of oregano EO and its single substances. In particular, carvacrol and thymol are phenolic compounds with similar structures (carvacrol: 5-isopropyl-2-methylphenol; thymol: 2-isopropyl-5-methylphenol), and the antimicrobial activity of phenolic compounds has been investigated against several phyto- and human pathogens [14]. Generally, the single substances of oregano EO have a lipophilic nature and are able to interfere with membrane-catalyzed enzymes and with enzymes responsible for energy and protein production causing the cell death [13].

The bioherbicidal effects of *O. vulgare* EO could be also due to its composition of phenolic compounds such as thymol and carvacrol [38]. Furthermore, oregano EO was able to inhibit significantly the seeds germination and the roots and shoots growth of *Avena sterilis* L., *Datura stramonium* L., *Cucumis sativus* L. and *Lactuca sativa* L. [38]. Further research demonstrated that several plant EOs and their phenolic compounds, carvacrol, and thymol, have herbicidal effects on weed germination and seedling growth of various plant species. In particular, the bibliographic research stated that *O. vulgare* ssp. hirtum is an allelopathic plant and had showed a notable effect against some biosensor plants such as *Avena sativa* and *Lemna minor*. Hence, the ecological role of volatile terpenes in multiple ecological functions and in the phenomenon of allelopathy in case of oregano EO was confirmed [39].

## 4. Materials and Methods

### 4.1. Chemicals 

Dulbecco’s Modified Eagle Medium (DMEM) with 4.5 g/L glucose, dimethyl sulfoxide (DMSO), 3-(4,5-dimethylthiazol-2-yl)-2,5-diphenyltetrazolium bromide (MTT) were purchased from Sigma Aldrich (Milan, Italy). Fetal Bovine Serum (FBS), glutamine, penicillin-streptomycin, and Phosphate Buffered Saline (PBS) were purchased from Euroclone (Milan, Italy). All solvents and chemicals were of an analytical grade.

### 4.2. Plant Material

The aerial parts of *O. vulgare* were collected from populations growing wild in Marconia di Pisticci (Basilicata Region, Southern Italy). The plant was identified by Prof. Vincenzo De Feo and a voucher specimen, DFE203/2012, was deposited at the Herbarium in the Medical Botany Chair of the Salerno University. The representative homogeneous sample of the plant was collected during “balsamic time” corresponding to the flowering stage.

### 4.3. Isolation of the Volatile Oil

One hundred grams of aerial parts of *O. vulgare* were ground in a Waring blender and then subjected to hydrodistillation for 3 h according to the standard procedure described in the European Pharmacopoeia [40]. The yellow EO was solubilized in *n*-hexane, filtered over anhydrous sodium sulphate and stored under N_2_ at +4 °C in the dark, until tested and analyzed.

### 4.4. GC-FID Analysis

Analytical gas chromatography was carried out on a Perkin-Elmer Sigma-115 gas chromatograph (Pelkin-Elmer, Waltham, MA, USA) equipped with a FID and a data handling processor. The separation was achieved using a HP-5MS fused-silica capillary column (30 m × 0.25 mm i.d., 0.25 μm film thickness). Column temperature: 40 °C, with a 5 min initial hold, and then to 270 °C at 2 °C/min, 270 °C (20 min); injection mode splitless (1 μL of a 1:1000 *n*-hexane solution). Injector and detector temperatures were 250 °C and 290 °C, respectively. Analysis was also run by using a fused silica HP Innowax polyethylenglycol capillary column (50 m × 0.20 mm i.d., 0.25 µm film thickness). In both cases, helium was used as carrier gas (1.0 mL/min).

### 4.5. GC/MS Analysis 

Analysis was performed on an Agilent 6850 Ser. II apparatus (Agilent Technologies, Inc., Santa Clara, CA, USA), fitted with a fused silica DB-5 capillary column (30 m × 0.25 mm i.d., 0.33 μm film thickness), coupled to an Agilent Mass Selective Detector MSD 5973; ionization energy voltage 70 eV; electron multiplier voltage energy 2000 V. Mass spectra were scanned in the range 40–500 amu, scan time 5 scans/s. Gas chromatographic conditions were as reported in the previous paragraph; transfer line temperature, 295 °C.

### 4.6. Identification of the Essential Oil Components

Most constituents were identified by GC by comparison of their *Kovats* retention indices (Ri) [determined relative to the *t*_R_ of *n*-alkanes (C_10_–C_35_)], with either those of the literature [41,42,43,44] and mass spectra on both columns with those of authentic compounds available in our laboratories by means NIST 02 and Wiley 275 libraries [45,46] The components’ relative concentrations were obtained by peak area normalization. No response factors were calculated. 

### 4.7. Cytotoxicity Test

#### 4.7.1. Cell Cultures and Treatments

The human hepatocellular carcinoma cell line HepG2 and the human embryonic kidney HEK293 cells were cultured in DMEM (supplemented with 10% fetal bovine serum, 2 mM glutamine, 100 U/mL penicillin, and 100 μg/mL streptomycin) and maintained at 37 °C in a humidified atmosphere containing 5% CO_2_. The final DMSO concentrations in the cell cultures were no greater than 0.8%: these concentrations do not affect cell viability. DMSO treated cells were used as a control in all the experiments.

#### 4.7.2. Cell Viability Assay (MTT Test)

In order to investigate the putative cytotoxic effects of the oregano EO, the 3-(4,5-dimethylthiazol-2-yl)-2,5-diphenyl tetrazolium bromide (MTT) assay was carried out on HepG2 and HEK293 cells [45,46]. The MTT assay is based on the capability of living cells to convert this yellow water-soluble tetrazolium salt into insoluble purple formazan crystals by cleavage of the tetrazolium ring by active dehydrogenase enzymes. This water insoluble product can be solubilized by using organic solvents and the resulting colored solutions are spectrophotometrically measured, getting an absorbance directly proportional to the percentage of viable cells. Briefly, HepG2 and HEK293 cells were plated in triplicate in 96-well plates at a density of 1.5 × 10^3^ cells/well, and allowed to adhere for 24 h. The cultured cells were then treated with different concentrations of studied oregano EO (25, 50, 100, 200, 400, and 800 μg/μL) and of the single constituents (0.01, 0.02, 0.03, 0.06, 0.12, and 0.25 μg/μL for carvacrol and citral and 0.06, 0.11, 0.22, 0.45, and 0.90 μg/μL for limonene and thymol), and were incubated for 24 h at 37 °C. Control cells were incubated with a comparable amount of the vehicle (DMSO in DMEM medium). After discarding the medium from the wells, MTT solution (0.5 mg/mL in DMEM) was added to the cells, incubating for 4 h at 37 °C.

The formazan crystals were finally dissolved in DMSO:isopropanol (1:1) and 1% Triton solution. The absorbance was measured at a wavelength of 570 nm, with background subtraction at 630–690 nm, using a GLOMAX Multi detection System (Thermo scientific, Waltham, MA, USA). The untreated cells were considered 100% viable. The concentration that reduces the cell vitality to half is observed as IC_50_. i.e., ″half maximal inhibitory concentration″ is a measure of the effectiveness of a substance in inhibiting cell multiplication.

$$\mathrm{Percentage}\;\mathrm{of}\;\mathrm{cell}\;\mathrm{viability}=\frac{\left[\mathrm{Test}\;\mathrm{compound}\;\mathrm{OD}\;-\;\mathrm{Blank}\;\mathrm{OD}\right]}{\left[\mathrm{Negative}\;\mathrm{control}\;\mathrm{OD}\;-\;\mathrm{Blank}\;\mathrm{OD}\right]}\;\times \;100$$

#### 4.7.3. Observation of Morphological Changes 

The observation of morphological changes occurred in the tested cell lines were performed by using inverted phase contrast microscopy (Nikon Eclipse TS100, Nikon, Tokyo, Japan). HepG2 and HEK293 cell cultures were seeded in 12-well plates at a density of 2 × 10^5^ cells/well, and then were treated for 24 h with different concentrations of oregano EO and of its single main constituents. The calculated values of IC_50_ for hepatoma cells have been selected for treatment of both studied cell lines (HepG2 and HEK293) in the case of a morphology-observation test.

### 4.8. Antibacterial Activity Test

#### 4.8.1. Tested Bacterial Strains

Two G+ve bacterial strains were tested: *B. megaterium* ITM100 from the collection of “Istituto Tossine e Micotossine, Bari, Italy”, *B. megaterium* isolated from *Actinidia chinensis* (Act.). One G−ve bacterial strain *E. coli*. All tested bacterial strains were conserved in the collection of the School of Agricultural, Forestry, Food and Environmental Sciences of Basilicata University, Potenza, Italy.

#### 4.8.2. Bactericidal Assay

The antibacterial test was carried out by using the disc diffusion method of Bhunia et al. [47] on King B nutrient media [48]. A suspension of each tested strain was prepared in sterile distilled water adjusted at 10^8^ CFU/mL (OD ≈ 0.2 nm). Then, an aliquot of soft agar (0.7%) and prepared suspension (9:1; *v*/*v*) was prepared and 4 mL were added over each plate (diameter 90 mm) containing 10 mL KB media. Blank Discs (6 mm)-OXOID were placed on the plate surfaces. Fifteen µL of EO and each single substance at concentrations 25% and 50% (*v*/*v*) diluted in water and Tween 20 (0.2%) was carefully applied over discs. The bactericidal activity was estimated by measuring the diameter of inhibition zone (mm) formed around each treated point as compared to the control.

### 4.9. Phytotoxic Activity Test

A bioassay based on seeds germination and root/shoot elongation was carried out to evaluate the phytotoxic effect of the studied oregano EO on seeds *R. sativus* (commonly known as radish), *L. sativum* (commonly known as garden cress) and on seeds of two weed species *S. arvensis* (wild mustard) and *P. canariensis* (canary grass).

The seeds of radish and garden cress were purchased from Blumen srl (Piacenza, Italy), while wild mustard and canary grass was collected from wild plants. The seed-surface were initially sterilized by using 70% ethanol for 30 s and ten were immersed in distilled water for 15 min, and then 10 seeds were placed on each Petri dish (90 mm diameter) containing three filter paper layers (Sigma-Aldrich, Saint Louis, MO, USA) pre-treated with 7 mL of distilled water (-ve control) or 7 mL from each prepared concentration of oregano EO. The studied oil was dissolved in a mixture of water/acetone (99.5:0.5) and 0.2% soln. of Tween 20^®^, and then was prepared at the following doses (2.5, 1.25, 0.62, 0.25, 0.12, and 0.06 μg/mL).

The germination conditions were 23 ± 2 °C for 48 h with artificial photoperiod. Controls were performed with a water/acetone mixture. Seed germination was observed directly in petri dishes after 48 h from the incubation time, whereas the radical elongation was measured in centimeter after 120 h of incubation time. The whole phytotoxic assay was repeated 3 times with 3 replicates per each. The germination index was calculated using the formula:

G.I. % = [(S.G.t × R.E.t)/(S.G.c × R.E.c)] × 100

GI: germination index; S.G.t: average number of germinated treated seeds; R.E.t: average radical elongation for treated seeds; S.G.c: Average number of germinated seeds for control; R.E.c: average radical elongation for control.

Data are expressed as the mean ± SDs for germination, radicle elongation, and germination index. Data were analyzed using SPSS statistical program with Tukey test.

## 5. Conclusions

From the available literature, oregano EO and its main bioactive constituents seem to have a great potential as anticancer therapeutic agents especially thymol and carvacrol. In particular, the EO and its two constituents, thymol and carvacrol, explicated promising results against hepatocarcinoma cells (IC_50_), estimated to be 236, 289, and 48 mg/L, respectively. Conversely, they showed higher values of IC_50_ regarding the inhibition of health renal cells, which were estimated to be 310, 940, and 90 mg/L, respectively. Citral and limonene showed a cytotoxic effect (IC_50_) against renal health cells estimated to be 32 and 120 mg/L, as compared to 35 and 294 mg/L in the case of hepatocarcinoma cells. Therefore, these two substances are not recommended as potential anticancer therapeutic agents. Further studies will be carried out to explain the specific action mechanism(s) of the investigated single constituents. On the other hand, the potential toxicity and the exact side effects of the studied substance were rarely taken into consideration in the majority of related research. Therefore, future studies should be directed to obtain high-purified natural bioactive compounds from oregano and other aromatic plants and to study their possible potential toxicity on health human cells. On the other hand, mixtures of different compounds could show a higher efficiency than the single components, so the next studies should take into account this possibility with deep experiments using different percentages of main components of EOs to estimate the synergistic effect. Finally, the obtained results demonstrated that oregano EO and its main constituents have a significant antibacterial activity and a moderate phytotoxic effect.

## Figures and Tables

**Table 1:** Chemical composition of the essential oil of *O. vulgare*.

No.	Identified Substances	Ki ^(a)^	Ki ^(b)^	% ^(c)^	Identification ^(d)^
1	*α*-Pinene	921	1032	0.1	1,2,3
2	Sabinene	966	1132	t	1,2
3	1-Octen-3-ol	974	1154	0.1	1,2
4	Octan-3-one	981	1253	t	1,2
5	3-Octanol	990	1393	t	1,2
6	*α*-Phellandrene	997	1150	t	1,2,3
7	*α*-Terpinene	1010	1189	t	1,2,3
8	*p*-Cymene	1018	1269	t	1,2,3
9	Limonene	1022	1205	1.3	1,2,3
10	1,8-Cineole	1024	1213	0.1	1,2,3
11	(*e*)-*β*-Ocimene	1044	1262	t	1,2
12	*γ*-Terpinene	1054	1256	t	1,2,3
13	*cis*-Sabinene hydrate	1062	1556	0.3	1,2
14	Terpinolene	1083	1265	t	1,2
15	Methyl benzoate	1089		t	1,2
16	*trans*-Sabinene hydrate	1093	1474	t	1,2
17	*endo*-Fencol	1115	1120	0.1	1,2
18	3-Thujanol	1155		t	1,2
19	Borneol	1160	1719	t	1,2,3
20	Terpinen-4-ol	1173	1611	0.8	1,2,3
21	*γ*-Terpineol	1209	1718	0.7	1,2,3
22	*trans*-Carveol	1214	1845	t	1,2
23	Thymol methyl ether	1231	1607	0.9	1,2
24	Carvacrol methyl ether	1241	1975	1.4	1,2
25	Linalyl acetate	1253	1665	t	1,2
26	(*e*)-Citral	1270	1727	2.5	1,2
27	*trans*-Carvone oxide	1280		t	1,2
28	Thymol and Carvacrol	1283	21982239	74.8	1,2,3
29	*δ*-Elemene	1337	1476	0.1	1,2
30	*α*-Cubebene	1349	1466	0.1	1,2
31	Thymol acetate	1354	1867	0.1	1,2
32	Eugenol	1358	2186	t	1,2
33	Piperitenone oxide	1365	1983	t	1,2
34	Cyclosativene	1370	1492	0.1	1,2
35	*α*-Ylangene	1375	1493	0.2	1,2
36	*β*-Bourbonene	1384	1535	0.1	1,2
37	*β*-Elemene	1390	1598	0.1	1,2
38	(*e*)-*β*-Damascone	1413	1830	t	1,2
39	*trans*-Caryophyllene	1419	1612	0.4	1,2,3
40	*β*-Copaene	1428		0.2	1,2
41	*trans*-α-Bergamotene	1434	1568	0.1	1,2
42	*α*-Guaiene	1437		0.1	1,2
43	Aromadendrene	1442	1628	0.4	1,2
44	*α*-Humulene	1453	1689	0.4	1,2,3
45	*allo*-Aromadendrene	1458	1661	0.2	1,2,3
46	*cis*-Cadina-1(6),4-diene	1462		0.1	1,2
47	*γ*-Gurjunene	1474	1687	0.4	1,2
48	*γ*-Muurolene	1480	1704	0.4	1,2
49	Valencene	1493	1741	0.2	1,2
50	*α*-Muurolene	1498	1740	0.1	1,2
51	Germacrene A	1505	1499	1.1	1,2
52	*δ*-Amorphene	1511		0.2	1,2
53	*δ*-Cadinene	1523	1773	1.0	1,2
54	Zonarene	1530	1729	0.1	1,2
55	*α*-Cadinene	1535	1743	0.1	1,2
56	*α*-Calacorene	1540	1942	0.1	1,2
57	*β*-Calacorene	1561	1942	0.1	1,2
58	Germacrene-d-4-ol	1573	2069	0.1	1,2
59	(−)-Spatulenol	1574	2150	0.3	1,2,3
60	Caryophyllene oxide	1581	2008	0.3	1,2,3
61	*β*-Atlantol	1606		0.1	1,2
62	*α*-Muurolol	1640		0.2	1,2
63	Cubenol	1645	2080	0.1	1,2
64	*α*-Cadinol	1653	2255	0.4	1,2
65	Oplopanone	1734	2568	t	1,2
66	*Z*-Lanceol	1759		0.1	1,2
67	Khusinol acetate	1815		t	1,2
68	Cedranediol-8S-13	1896		0.2	1,2
69	Columellarin	1948		t	1,2
70	Ethyl hexadecanoate	1989		t	1,2
71	Methyl linoleate	2106		t	1,2
	Total			91.4	
	Monoterpene Hydrocarbons			1.7	
	Oxygenated Monoterpenes			4.2	
	Sesquiterpene Hydrocarbons			5.8	
	Oxygenated Sesquiterpenes			1.5	
	Phenolic compounds			77.2	
	Other compounds			0.1	

^(a)^ Kovats retention index determined relatively to the *t*_R_ of a series of *n*-alkanes (C_10_–C_35_) on HP-5 MS column. ^(b)^ Kovats retention index determined relatively to the *t*_R_ of a series of *n*-alkanes (C_10_–C_35_) on HP Innowax. ^(c^^)^ t = trace (<0.1%). ^(d)^ 1 = Kovats retention index, 2 = mass spectrum, 3 = co-injection with authentic compound.

**Table 2:** Evaluation of cytotoxicity of main single constituents of oregano EO against HepG2 cells.

Single Substances	Trend-Line Equation	IC_50_ (mg/L) ± SDs
Limonene	Y = 24.51X − 12.89	294.3 ± 10.5
Thymol	Y = 27.28X − 26.70	289.5 ± 6.6
Carvacrol	Y = 19.90X − 18.25	48.3 ± 6.7
Citral	Y = 20.47X − 26.49	35.5 ± 4.5

Where: Y is the cell viability (%), X is the tested concentration (mg/L).

**Table 3:** Evaluation of cytotoxicity of main single constituents of oregano EO against healthy renal cells.

Single Substances	Trend-Line Equation	IC_50_ (mg/L) ± SDs
Thymol	Y = 14.56X + 35	939.8 ± 29.6
Limonene	Y = 12.61X − 13.80	120.3 ± 12.0
Carvacrol	Y = 8.91X + 27.96	90.5 ± 9.9
Citral	Y = 6.58X + 23.41	32.0 ± 5.1

Where: Y is the cell viability (%); X is the tested concentration (mg/L).

**Table 4:** Antibacterial activity of *O. vulgare* EO and its main active constituents.

Diameter of Inhibition Zones (mm)				
Treatments	Conc (%)	G+ve		G−ve
		*B. meg* Act.	*B. meg* 100	*E. coli*
Oregano EO	50	40.0 ± 3.0a	43.0 ± 3.2a	22.0 ± 2.4a
	25	28.0 ± 1.5b	31.0 ± 1.9b	15.0 ± 1.3b
Carvacrol	50	45.0 ± 4.0a	45.0 ± 2.8a	20.0 ± 2.2a
	25	30.0 ± 2.2b	35.0 ± 2.1b	11.0 ± 1.7b
Thymol	50	30.0 ± 2.7b	35.0 ± 3.2b	20.0 ± 1.9a
	25	20.0 ± 1.4c	0.0 ± 0.0e	0.0 ± 0.0c
Limonene	50	22.0 ± 3.5c	27.0 ± 1.7c	15.0 ± 1.1b
	25	15.0 ± 2.7d	17.0 ± 1.1d	0.0 ± 0.0c
Citral	50	45.0 ± 25a	45.0 ± 2.7a	25.0 ± 2.7a
	25	40.0 ± 1.8a	40.0 ± 1.8a	15.0 ± 1.4b

Where: *B. meg* Act.: *Bacillus megaterium* actinidia; *B. meg* 100: *B. megaterium* from ITM collection; and *E. col*: *Escherichia coli*. Values are recorded as the mean of growth inhibition percentage ± SDs. Values followed by the different letter in each vertical column for each strain are significantly different according to Tukey B test at *p* < 0.05. Data were obtained from three replicates ± SDs.

**Table 5:** Phytotoxic activity of oregano EO against seed germination and radical elongation (cm). Data of seed germination are expressed in number, radical elongation are expressed in cm and germination index are expressed as %.

	Doses	G.S. ± SD	R.E. ± SD	G.I. %
***Sinapis Arvensis***	Control	6.9 ± 0.7	1.4 ± 0.5	100.0 ± 0.0
	0.062 µg/mL	7.7 ± 0.6	1.3 ± 1.2	103.6 ± 4.6
	0.125 µg/mL	6.3 ± 0.4	1.7 ± 1.0	110.9 ± 6.0
	0.25 µg/mL	7.7 ± 0.7	1.9 ± 1.0	151.4 ± 8.0
	0.625 µg/mL	7.0 ± 0.9	1.8 ± 0.8	130.4 ± 6.0
	1.25 µg/mL	6.7 ± 0.4	1.5 ± 1.2	104.0 ± 4.0
	2.5 µg/mL	6.7 ± 0.5	1.0 ± 0.8	69.4 ± 6.7 *
***Phalaris Canariensis***	Control	6.9 ± 0.7	1.4 ± 0.5	100.0 ± 0.0
	0.062 µg/mL	7.7 ± 0.6	1.3 ± 1.2	79.5 ± 5.4 **
	0.125 µg/mL	6.3 ± 0.4	1.7 ± 1.0	107.6 ± 9.7
	0.25 µg/mL	7.7 ± 0.7	1.9 ± 1.0	83.7 ± 6.4
	0.625 µg/mL	7.0 ± 0.9	1.8 ± 0.8	95.3 ± 4.6
	1.25 µg/mL	6.7 ± 0.4	1.5 ± 1.2	100.5 ± 5.4
	2.5 µg/mL	6.7 ± 0.5	1.0 ± 0.8	83.7 ± 3.7
***Lepidium Sativum***	Control	6.2 ± 1.0	0.8 ± 0.3	100.0 ± 8.0
	0.062 µg/mL	6.0 ± 1.0	0.8 ± 0.3	96.8 ± 6.0
	0.125 µg/mL	4.3 ± 1.0 *	0.4 ± 0.1	34.7 ± 4.2 *
	0.25 µg/mL	4.3 ± 0.5 *	0.5 ± 0.2	43.34 ± 5.7
	0.625 µg/mL	5.3 ± 0.9	0.6 ± 0.2	64.1 ± 8.0
	1.25 µg/mL	5.3 ± 0.9	0.7 ± 0.1	74.8 ± 6.1
	2.5 µg/mL	5.3 ± 0.7	0.7 ± 0.3	74.8 ± 4.9
***Raphanus Sativus***	Control	6.9 ± 1.0	2.6 ± 0.8	100.0 ± 8.0
	0.062 µg/mL	3.3 ± 1.2	2.1 ± 1.7	67.3 ± 4.5
	0.125 µg/mL	2.7 ± 0.8 *	1.1 ± 0.3 *	28.9 ± 6.2 *
	0.25 µg/mL	5.7 ± 0.4	1.5 ± 0.7	83.1 ± 8.4
	0.625 µg/mL	2.3 ± 1.0 *	1.5 ± 0.4	33.5 ± 5.4 *
	1.25 µg/mL	3.3 ± 0.8	1.6 ± 0.1	51.3 ± 4.5
	2.5 µg/mL	3.3 ± 0.9	1.6 ± 0.4	51.3 ± 3.1

Where G.S.: seed germination; R.E.: radical elongation; G.I.: germination index. Values are means ± SDs “standard deviations of 3 replicates”. Data were analyzed using SPSS statistical program with Tukey test, significant differences compared to control: *p* < 0.05 (*), *p* < 0.01 (**).
